# Hepatitis C Virus in North Africa: An Emerging Threat

**DOI:** 10.1155/2016/7370524

**Published:** 2016-08-16

**Authors:** Mohamed A. Daw, Abdallah El-Bouzedi, Mohamed O. Ahmed, Aghnyia A. Dau, Mohamed M. Agnan

**Affiliations:** ^1^Department of Medical Microbiology, Faculty of Medicine, Tripoli University, P.O. Box 82668, Tripoli, Libya; ^2^Department of Laboratory Medicine, Faculty of Biotechnology, Tripoli University, P.O. Box 82668, Tripoli, Libya; ^3^Department of Microbiology and Parasitology, Faculty of Veterinary Medicine, Tripoli University, P.O. Box 13662, Tripoli, Libya; ^4^Department of Surgery, Tripoli Medical Centre, Faculty of Medicine, Tripoli University, P.O. Box 82668, Tripoli, Libya; ^5^Department of Pharmacology, Faculty of Medical Technology, Algabal Algarbi University, P.O. Box 3321, Nalut, Libya

## Abstract

Hepatitis C virus is a major public health threat associated with serious clinical consequences worldwide. North Africa is a unique region composed of seven countries that vary considerably in the predisposing factors to microbial diseases both historically and at the present time. The dynamics of HCV in the region are not well documented. The data are both limited and controversial in most of the countries in the region. In North Africa, the epidemiology of HCV is disparate and understanding it has been hampered by regional “epidemiological homogeneity” concepts. As the dynamics of HCV vary from country to country, context-specific research is needed. In this review, we assess studies performed in each country in the general populations as well as among blood donors and groups exposed to the HCV infection. The reported prevalence of HCV ranges from 0.6% to 8.4% in the Maghreb countries and is predominated by genotype 1. In the Nile valley region, it ranges from 2.2% to 18.9% and is dominated by genotype 4. In North African countries, HCV seems to be a serious problem that is driven by different vectors even in different geographical locations within the same country. Efforts should be combined at both the national and regional levels to implement efficient preventive and treatment strategies.

## 1. Introduction

Hepatitis C virus (HCV) is one of the most important viruses. The dynamic nature of the virus, its transmission by various vectors, and the diversity of the subtypes are reflected in the epidemiology of hepatitis C infection. The global prevalence of anti-HCV has been estimated at 2.0% (1.7–2.3%) among adults and 1.6% (1.3–2.1%) for all ages. These percentages mean that there are about 115 million people infected with HVC, of whom about 104 million are adults [1]. The World Health Organization (WHO) recognizes viral hepatitis as a global health challenge from which no country, rich or poor, is spared. As hepatitis C virus infection is not preventable by vaccination, WHO urges member states to take action to improve surveillance, prevention, and access to screening and treatment at the national and regional levels [2].

Hepatitis C virus infection is a major public health concern particularly in African countries, which have the highest prevalence rates of HCV in the world (1–26%) [3]. In Africa over 28 million people are chronically infected with HCV and it is difficult to speculate about current and future trends [4]. The prevalence of HCV varies from one African country to another. Comparison of the epidemiological status in different countries is difficult due to variations in diagnostic procedures, multiplicity of definitions of infection, and the use of different methods, as well as the time at which the epidemiological research was done [5].

Due to the great diversity in prevalence and modes of transmission, accurate epidemiological information on HCV infections is urgently needed to guide national and regional plans for prevention, treatment, and reduction of complications of the infection [6]. Accurate data are scarce for many regions, particularly parts of sub-Saharan and North Africa, partly because the public health importance of HCV infection has been recognized only recently [7].

In North Africa, accurate assessment of the burden of hepatitis C infection is hampered by the lack of adequate surveillance and by poor resources for proper data collection and management. Despite the geographic proximity of these countries and longstanding interaction between them, the prevalence and complications of HCV are greatly different between them. According to current estimates, the lowest prevalence of the virus is in Libya (0.9–1.6%) and the highest is in adjacent Egypt (12.5–26.6%) [8]. This review aims to navigate the reported epidemiology of HCV in the region of North Africa to describe the prevalence of HCV in each country, highlight the predisposing factors, and outline the strategies needed to halt the spread of HCV and its consequences.

## 2. Geoepidemiological Considerations

The North African region houses about 23% of the African population. It is divided into two subregions, the Maghreb (Algeria, Libya, Morocco, Tunisia, and Mauritania) and the Nile valley (Egypt and Sudan). Egypt is a transcontinental country because its Sinai Peninsula lies in western Asia. North Africa also includes a number of Spanish possessions (Ceuta and Melilla, part of coast of Morocco). Despite the similarities between these countries in environment, population genetics, social customs, and habits, they differ greatly in the infrastructure of their healthcare systems, per capita income, level of urbanization, and degree of poverty. Their populations vary from poor nomadic Bedouins scattered in the Sahara areas of Mauritania, Algeria, Morocco, and Sudan to sheltered valleys in the Atlas Mountains, the Nile Valley, and delta, up to urbanized cities on the Mediterranean coast such as Tripoli and Benghazi. Epidemiological information on HCV among these populations is fragmentary and obtaining more information is hampered by poverty, ignorance, and more recently uprisings and conflicts [9].

**Figure 1:**
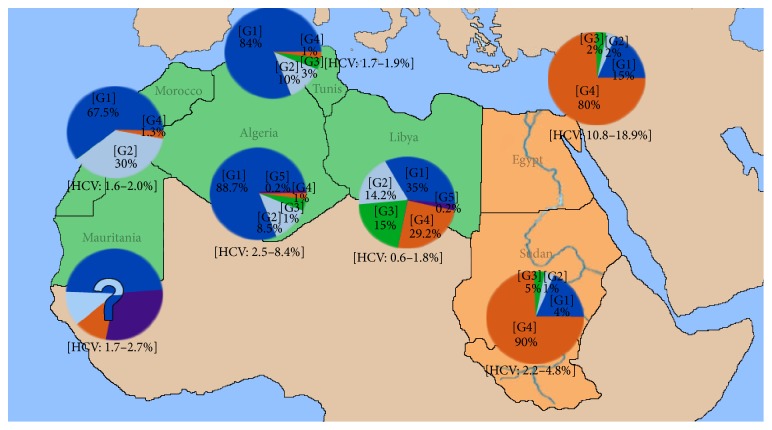
Geographic distribution of HCV genotypes among HCV-infected individuals in North Africa. G1, G2, G3, G4, and G5 refer to the respective genotypes of HCV.

With the exception of Libya, Egypt, and Morocco, North African countries lack adequate surveillance studies on hepatitis C virus to enable them to take evidence-based policy decisions [10–12]. Their data are outdated, aggregated, or limited to small specific populations and affected by selection bias because most of their studies are generally based on risk groups or blood donors, and information on children and the elderly is generally not included [10, 13]. In North Africa, predisposing factors and epidemiological information on HCV vary significantly from one country to another. Moreover, there is considerable variation in the economic and demographic situations in these countries (Table 1). Therefore, the assumption of “epidemiological homogeneity” cannot be easily adopted because it may lead to inaccurate estimates of HCV seroprevalence in these countries [14]. Hence then, the epidemiological features of HCV in each country should be considered separately. The region's contribution to the global literature on HCV remains relatively small, though the number of publications from Egypt, Libya, and Morocco has increased.  Figure 1 shows the prevalence of HCV and genotype distribution in all the North African countries.

## 3. Epidemiology of HCV in the Maghreb Region

### 3.1. Libya

Libya is the richest country in North Africa. It is also the second largest and has a small population. HCV is well documented in this country and major studies were carried out on all aspects of HCV infection over the last 20 years [15]. The largest comprehensive national study in Asia and Africa was carried out in Libya and covered over 1% of the total population [11]. The overall prevalence rates of HCV in males and females were similar (1.1% and 1.3%, resp.). The mean age of anti-HCV-positive individuals was 31.7 ± 18.4 years in females and 35.6 + 20.9 years in males. The mean age of HCV-positive individuals was significantly higher than that of anti-HCV negative individuals for both males (almost 10-year difference) and females (7-year difference). HCV was more prevalent among single and younger individuals, and about 40% of HCV patients were less than 30 years old. The prevalence of HCV was higher among illiterate individuals (3.1%) than among literate persons (0.9–1.1%). Hepatitis C was most prevalent among intravenous drug users (7.4%) and less prevalent but still substantial in those undergoing blood transfusion (2.7%), surgical operation (2.3%) or hospital admission (1.9%).

The prevalence of HCV infection in Libya varied widely between hemodialysis centers from 0% to 35.9%. Compared to seronegative patients, seropositive patients were younger and had been receiving dialysis for substantially longer. Follow-up showed that 7.1% of the dialysis patients seroconverted during the first year of dialysis [16].

A total of 20 discrete genotypes and subtypes were identified countrywide in the Libyan population, and their frequencies ranged from 11.5 to 0.3% among Libyan HCV patients. Genotype 4 was the most frequent among all regions (19.7–40.5%), reaching the highest value in eastern region (27%), followed by genotype 1 which was more prevalent in the southern (49.3%) and western (40.0%) regions. Genotype 3 was more prevalent in Tripoli area (21.3%) and the eastern regions (15.9%), while genotype 2 was common in the northern (23.6%) and southern (22.5%) regions [17, 18]. The frequencies of these genotypes were significantly associated with the demographic and risk factors involved. Transmission by intravenous drug use (IVDU) has become more frequent in Libya and is associated mostly with genotype 1 (49.2%) and genotype 3 (32.6%).

### 3.2. Tunisia

Tunisia has the smallest area among the Maghreb countries and is bounded by the two largest countries in the region, Libya and Algeria. Different studies were carried out on HCV infection in Tunis. Two decades ago, Triki et al. studied the prevalence of hepatitis B and hepatitis C and delta virus infections in Tunis in a population of mainly male military recruits aged 20–25 years [19]. The overall prevalence of HDV/HCV coinfection was 17.7% but it was low for HCV alone (2.7%). In 2005, another study reported a prevalence of HCV of 1.7% in the general population and great heterogeneity in geographical distribution. HCV was particularly more prevalent in the northwestern region of the country than anywhere else. But there was no difference in positivity according to gender or to living in rural versus urban areas; the only significant risk factor was advanced age [20]. Similar results were reported for blood donors and diabetic patients, in whom prevalence of anti-HCV antibodies varied between 0.5% and 1.8% [21]. However, these studies suffer from lack of specificity and were confined to certain populations. They did not accurately mirror the status of HCV within the country. A recent seroprevalence study of transfusion-transmitted infections in first-time volunteer and replacement donors in Tunisia showed that HCV, according to mathematical adjusted model, reached 1.9% (95% CI = 0.9–4.1 *P* = 0.11) [22]. Hence, further studies are needed to assess the actual burden of CV infection in Tunisia.

The prevalence of HCV infection among Tunisian dialysis patients was reported to be high, reaching up to 51%. There was a close correlation between the number of anti-HCV-positive patients and the duration of dialysis therapy [23, 24]. This indicates the nosocomial transmission of HCV in dialysis units where the number of infected patients is high and where the management of material does not take into account the patient's viral status.

The reported genotype patterns among Tunisians vary substantially from one study to another. Subtype 1b was the most common (79%), whereas types 1a, 2a, 2b, 3a, and 4a occurred much less frequently. Furthermore, subtype 4k seems to have disappeared in Tunis city in conjunction with the emergence of a new subtype of HCV4 [25, 26].

### 3.3. Algeria

Algeria is the largest country in North Africa and its population lives a range of traditional/rural or modern/urban lifestyles. Data on the epidemiology of HCV in Algeria are scarce. Official data point to a current hepatitis C epidemic [27], and the Algerian Ministry of Health estimated that the prevalence of HCV infection had reached 2.5% [28, 29].

Seroprevalence rates of HCV reaching 53% were reported in patients undergoing hemodialysis and 31.6% among hemophilia patients in Algeria. This situation is plausibly connected with nosocomial transmission and occupational exposure to HCV among healthcare workers [29].

In a rare retrospective study, Rouabhia et al. investigated hepatitis C virus markers in 739 diabetic and 580 nondiabetic patients attending the internal medicine department of the University Hospital Center of Batna in Algeria [30]. Anti-HCV seropositivity was 17.5% in diabetic patients and 8.4% in nondiabetic patients (*P* < 0.01). However, after adjustment for age, this difference is statistically significant only in patients aged 40–65 years (22.2% versus 9.3%, *P* = 0.024). Despite the ongoing controversy being whether diabetes mellitus is a risk factor for HCV infection or HCV is a risk factor for type 2 diabetes mellitus, the prevalence of HCV among healthy Algerian in this age group is indeed high (8.4–9.3%) [30].

In Algeria, HCV genotype 1 was the most frequent (88.7%), followed by genotypes 2 (8.5%), 4 (1.1%), 3 (0.9%), and 5 (0.2%). The genotype distribution was related to age and region. Genotype 1 was significantly less frequent in the ≥ 60-year age group than in younger people (OR = 0.2; 95% CI: 0.1–0.5, *P* < 0.001) [27, 30]. Furthermore, genotype 1 was more frequent in the central part of the northeastern region of Algeria than elsewhere.

### 3.4. Morocco

Morocco is the western bounding arm of the Maghreb region, facing both the Atlantic Ocean and the Mediterranean Sea. Different studies were carried out on HCV in Morocco both in the general population and on higher risk groups. Early studies estimated that the prevalence of HCV was 1.93% in the general population and 1.08% in blood donors [31]. A recent nationwide cross-sectional survey carried out in 100 major Moroccan regions over a period of six years showed that the overall prevalence of HCV infection in the general population was 1.58%, and it was lower among blood donors [10]. The prevalence was higher among males ≤ 30 years old. Factors significantly associated with HCV infection were increasing age, dental treatment, use of glass syringes, and history of surgery. Emerging data suggest that differences in anti-HCV prevalence may exist between the northern and southern regions in Morocco [32]. HCV is a major problem in hemodialysis centers in Morocco. A multicenter study covering different Moroccan dialysis centers found that the prevalence varied from 11% to 91% [33].

Practitioners of traditional medicine and barbers play an important role in the spread of HCV in Morocco. A survey of anti-HCV antibodies among barbers and their clients indicated that the prevalence rate hovered above 5%, probably because of unsanitary conditions [34]. This problem could also exist in Libya, where most of the hairdressers are Moroccans.

Drug addiction is a serious problem in Morocco and has been considered to be a “male-associated habit” because the overwhelming majority of drug addicts are young males who are either single or divorced. HCV seroprevalence is high among this group, reaching up to 60% [35].

The commonly reported HCV genotypes in Morocco were genotypes 1 (46%) and 2 (40%), followed by genotypes 3 and 4. Among intravenous drug users, genotype 1 accounted for 65% of the cases, followed by genotype 3 (26%) and genotype 4 (10%) [36].

### 3.5. Mauritania

Mauritania is a Saharan country characterized by a very high prevalence of viral hepatitis that poses a serious public health problem. In a recent coherent study, up to 20% of consulting patients and pregnant women or blood donors have HBV, and up to 33% have HDV [37]. Despite the limited published data on HCV in Mauritania, HCV seems to be more prevalent in Mauritania than in other Maghreb countries. Mansour et al. conducted a comprehensive prospective study on 1966 individuals and showed that the overall prevalence of HBsAg was 18.3%. The prevalence was significantly higher in males (24.4%) than in females (13.8%) [*P* < 0.001; OR: 2.04 (1.46–2.85), *P* < 0.001]. It varied significantly among the different ethnic groups: 22.7% in white Moors, 19.7% in black Moors, and 12% in African ethnicities [*P* < 0.025, OR: 0.47 (0.26–0.82), *P* < 0.008] for the comparison between White Moors and other African ethnic groups [38, 39]. The characteristics of individuals positive for HBsAg strongly suggest healthcare-associated transmission, intrafamilial transmission, sexual transmission, more frequently a history of hospitalization, and transmission through iatrogenic, medical, or paramedical procedures.

In 2000, 2854 healthy blood donors were screened for HCV antibodies at the National Hospital of Nouakchott, and the prevalence of HCV was found to be 2.7%, but no risk factors were studied [40, 41]. However, it has been speculated that the prevalence of HCV in Mauritania may be as high as 10.7%, similar to that in West African countries [8].

## 4. Epidemiology of HCV in the Nile Valley Region

HVC prevalence in the Nile Valley region has unique features and specific epidemiological characteristics that have implications for the prevention and future prospects of HCV in the region.

### 4.1. Egypt

Egypt is confronted with a huge HCV infection problem that distinguishes it from the rest of North Africa. It has the highest prevalence of HCV in the world, and HCV infection and its complications are among the leading public health challenges in the country [40]. The epidemiological and clinical status of HCV in Egypt is well known and different studies were carried out to examine the different aspects of this epidemic [41]. Data on HCV in Egypt are diverse and vary greatly from one study to another. Nevertheless, they all reach the same conclusion that the prevalence of HCV is very high among all groups and populations [42, 43].

A major survey conducted in 2008 reported a HCV prevalence of 14.7% in a nationally representative sample of 11,126 Egyptians aged 15–59 years. The infection rate increased steadily with age. It was nearly zero in children under the age of 9 years, 5% among those aged 30–39 years, and 10% among those aged ≥ 50 years [12]. The study also showed that rural villages had a higher prevalence than urbanized cities [44]. Studies conducted in the Nile delta region, Assuit and Benha, showed a higher prevalence among all sectors studied, including pregnant women and children [45].

Overall, the average HCV prevalence among risk groups is even higher. It was reported to be 38% among schistosomiasis patients, 63.0% among intravenous drug users, and 46.1–100% among hemodialysis patients. Population groups at an intermediate risk of exposure include diabetic patients, hospital outpatient attendees, hospitalized patients, household contacts of index cases (HCV-positive cases), patients with sexually transmitted infections, patients with periodontal disease, prisoners, and healthcare workers [46, 47].

HCV genotype 4 (subtype 4a) predominates in Egypt and is responsible for > 90% of the infections; the rest of the infections are due to genotype 1 (1b, 1g) and genotype 3 (3a) [48]. Recent data show that the overall prevalence of HCV in Egypt is declining as those who were initially infected are aging and dying. However, the disease burden of HCV and associated costs will continue to grow due to the increasing number of individuals developing advanced liver disease and dying from liver pathologies related to HCV rises [49].

### 4.2. Sudan

Sudan is the largest country in the Nile valley with a land mass about the size of Europe. It has been engaged in an ongoing civil war for 20 years, and one of the consequences is about 4 million refugees [50]. The Sudanese community is characterized by great social and demographic diversity reflected in the epidemiology of microbial diseases in the country. Studies on HCV in Sudan are few and lack specific national goals. The few studies on HCV infection in Sudan demonstrated a seroprevalence ranging from 2.2% in the Gezira state, in which schistosomiasis is endemic, to 4.8% in patients with schistosomal periportal fibroses [51]. The prevalence of HCV infection among asymptomatic male Sudanese blood donors was 4.4%, but females do not donate blood in Sudan. Other studies reported prevalence rates of 3% and 1.5% in southern and northern regions of Sudan, respectively. Furthermore, HCV transmission was evident in healthcare settings, and occupational risk is expected to be high. In presurgery screened patients in Khartoum, central Sudan, prevalence of HCV was 2% [52].

Unprotected sexual activity (20%) was the most apparent predisposing risk factor for HCV seroreactors, followed by razor sharing (13.3%), parenteral drug injection (10%), tattooing, and surgical procedures. The highest prevalence of HCV infection in Sudan was noted in patients with end-stage renal disease who were on regular hemodialysis (seroprevalence of 66.7%) [52, 53]. Major risk factors for infection were longer duration of dialysis, dialysis in multiple centers, and an age over 30 years. Genotype 4 was the most frequently isolated genotype among HCV-positive patients in Sudan [53].

Studies on the HCV status among intravenous drug users and HIV patients in Sudan are lacking. Recently, Sudanese researchers raised the importance of this issue, particularly as Sudan borders nine African countries on the east and the south that have some of the highest HIV-1 infection rates in the world [54]. The HCV status in Sudan is not well documented, particularly for high risk groups and in healthcare settings. Further studies are urgently needed, including but not limited to population-based studies that are representative of entire communities, and a national cooperative registry system should be established.

## 5. Vectors of HCV Transmission in the North Africa

### 5.1. Drug Trafficking

Drug trafficking poses specific problems for North African countries, and it is exacerbated by the geographical location and the vast area of the region. Morocco is the world's foremost producer of cannabis resin and remains the main source of the drug for the consumer markets in Western Europe; the largest seizure in 2007 was in Mauritania [55, 56]. Studies from the Middle East and North Africa regions indicate that after Iran, the largest numbers of people injecting drugs are in Egypt and Algeria [57]. Illicit drug injection is also a significant route of HIV transmission in Libya and Tunisia, as well as in Sudan. Cocaine use is reportedly increasing in these countries, but cocaine injection has not been reported. Use of noninjectable drugs has not been shown to be linked to the transmission of HIV or HCV. Information on HCV prevalence among people who inject drugs is not available for most countries in the region, but, where reported, the data reveal high levels of HCV infection [58]. Furthermore, men who have sex with men and people who inject drugs are both highly criminalized populations in region and they are more affected by HCV and HIV than the rest of the population [59].

### 5.2. Urbanization Level

Urbanization has contributed to an overall improvement of health status [60]. Different studies have shown that the level of urbanization could influence the prevalence of HCV in the North African region. Libya, which is considered an urbanized country, has the lowest HCV prevalence. The other countries are mainly rural and are not expected to pass the urban tipping point before 2050 [61]. Sociodemographic studies comparing rural with urban Egyptian populations have shown a higher prevalence among rural populations [62]. Blood donors and children from rural areas had a higher prevalence of HCV than those from urban areas [63]. Similar results were reported for hepatocellular carcinoma in rural and urban areas [48]. The same pattern was reported in Tunisia, Algeria, and Morocco. HCV prevalence was significantly higher in the northwestern region of Tunisia and the suburban area of Tunis than in the northern region. In Algeria, HCV infection was strongly associated with living in remote mountains or desert regions as compared to living in urbanized environments along the Mediterranean coast [27]. In Morocco, being a rural resident was found to be strongly associated with HCV infection. In Mauritania and Sudan, the vast majority of the population is composed either of rural dwellers or Bedouins even within the surroundings the capital cities. This could be attributed to the habits and economic status of this major subpopulation. Lack of sanitary services, illiteracy, traditional medicine practice, and occupational hazards and not adopting safe medical practices may contribute to higher levels of HCV infection. However, further studies are needed to verify this assumption.

### 5.3. Medical Practices and Personal Habits

Hemodialysis, blood transfusion, and practices associated with hospital care, combined with social or personal habits and other community-associated factors, are among the most important risk factors for HCV in North African countries. Mortality and morbidity due to infections associated with such factors are expected to be high, and the adjusted hazard ratio for patient death may reach 2.3 [64]. The prevalence of HCV infections observed in blood products and dialysis patients in these countries are much higher than in the general population. This situation is worsened in areas of chronic conflict, such as Sudan and the Sahara region, as well as in regions newly gripped by war, notably Libya, in which blood supplies were safer and more secure before the 2011 uprising. Tunisia and Libya experienced stock-outs during their recent uprisings both for blood screening reagents and for access to new antiretroviral drugs. The impact of these conflicts on the healthcare system should be evaluated [65]. Healthcare workers should be sufficiently informed about the risk of acquiring HCV via sharp injuries and other nosocomial routes. Habits and cultural factors that may influence the spread of HCV in North African countries include male and female circumcision, particularly in Egypt, Sudan, Mauritania, and Morocco. Hijiama (bloodletting) done by informal practitioners, tattooing, folk body piercing and threading, sharing hygiene tools and sharp items, and the use of communal barbers may be considered as risk factors for HCV, particularly among rural dwellers [21, 34]. Education and public awareness campaigns are needed to teach the populations about the risks involved.

### 5.4. HCV/HIV Coinfection

There is little information on HCV/HIV coinfection in North African countries. The paucity of information on injecting drug use and HIV in these countries could be attributed to the reluctance to commission research or publicize information on these two highly stigmatized issues, and such avoidance could mask the true extent to which people who inject drugs are affected by HIV [66]. The reported prevalence rates of HCV/HIV coinfection vary depending on the route of transmission. Morocco is the only country in the region that has reliable surveillance programs for HIV. Prevalence of HIV/HCV coinfections in Morocco was reported to be about 20% among injecting drug users and sex workers, but it was 10.6% among patients of different socioeconomic backgrounds [35].

Women have already overtaken men in their contribution to the HIV epidemic in Sudan, Algeria, and Tunisia, where the number of AIDS cases is higher in women than in men. In Morocco, the situation is becoming increasingly feminized [67]. A study carried on 4220 female sex workers aged 15–49 years recruited from 14 states in Sudan indicated that the prevalence of HIV ranged from 4.4 to 23.9%, with 6% coinfected with HCV [67]. In Tunisia, the prevalence rate of anti-HCV positivity was 40% among HIV-infected patients, of whom 78% were injecting drugs. In Libya, over 90% of HIV cases are attributable to injecting drugs. A population-based study in nine districts in Tripoli showed that the average prevalence rates of HIV reached 0.2%, whereas HBV and HCV rates reached 3.7% and 0.9%, respectively [68]. In Egypt, the rate of HIV infection among female sex workers was much higher (up to 36.3%) than among women who do not sell sex [69]. Furthermore, a randomized detailed survey in 2010 found that 6.8% of intravenous drug users in Cairo and 6.5% in Alexandria were infected with HIV [70]. HCV coinfection among these populations ranged from 37 to 86% in Egypt. The coemergence of HCV in HIV infected patients has become a serious problem in the North African region: effective preventive and monitoring programs have to be implemented [70].

### 5.5. Miscellaneous Factors

Various other factors could contribute to the epidemicity of HCV in North Africa, including imprisonment, alcoholism, and sex practices, which are stigmatized socially and culturally. The situation is exacerbated by lack of human rights, injustice, and the recent political uprisings in this region. More than a third of all prisoners and more than 80% of injecting drug users were positive for antibodies to hepatitis C virus in North African countries [13]. Libya is the only country in the region for which there is an available estimate of the number of prisoners with a history of injecting drug use (approximately 60%) [71]. Alcohol drinking and female sex work are other factors in North African countries. Alcohol consumption accelerates the course of chronic hepatitis C. In Mauritania and Libya alcohol consumption and prostitution are banned according to Islamic laws. Recorded alcohol consumption rates are variable across North Africa. Sudan has low levels of alcohol consumption. On the other hand, Morocco, Egypt, Algeria, and Tunis have higher consumption rates [56, 72]. HCV and other associated sexually transmitted diseases are rarely studied in North African countries, leaving only hypothetical speculation. The only formulated and well planned study was done in Sudan [67]. Biobehavioral surveys using respondent-driven sampling were carried out among female sex workers in the capital cities of 14 states in Sudan in 2011-2012. The findings point to a high burden of sexually transmitted diseases in female sex workers [67]. The highest prevalence of HCV was found in the western zone (2.6% and 5.1% at two sites). However, there is little information on the prevalence of HCV among injection drug users. Hence, further studies are needed.

## 6. Consequences of HCV in North Africa

**Figure 2:**
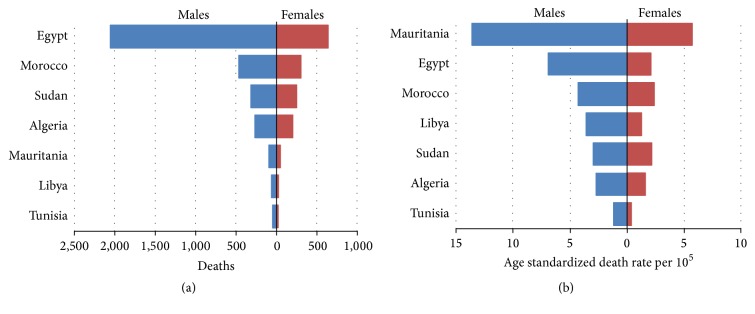
Mortality from HCV-associated hepatocellular carcinoma in North Africa.

Hepatitis C infection has been implicated in the development of hepatocellular carcinoma (HCC) in North African countries. A multicenter study of the risk factors for hepatocellular carcinoma was carried out in cooperation between Morocco, Algeria, and Tunisia. The study showed that over 60% of HCC patients were positive for anti-HCV, though only 17.9% and 19% were positive for HBsAg or had diabetes, respectively [73], A33-fold higher in HCV infection comparable by just 10-fold for HBV. However, the association was mainly for HCV genotypes 1 and 2. Egypt had by far the highest burden of deaths from HCV-associated HCC, and 63.3% of all HCV-associated HCC deaths occurred in Egypt (Figure 2(a)), followed by Morocco, Sudan, and Algeria. The figures were lower for Libya and Tunisia. Mauritania and Egypt also had the highest age-standardized rates deaths (ASDR) for HCV-associated HCC in both males and females (Figure 2(b)) [74]. There is no easy and effective treatment for HCC, so preventing the transmission of hepatitis viruses is the most important step to reduce the risk of HCC in these countries. Extrahepatic diseases also cause morbidity among HCV-infected individuals. Patients with chronic hepatitis C have a higher risk of developing diabetes, thyroid disease, lichen planus, and an array of neuropsychiatric disorders [75, 76]. These conditions are rarely studied in the region of North Africa.

**Figure 3:**
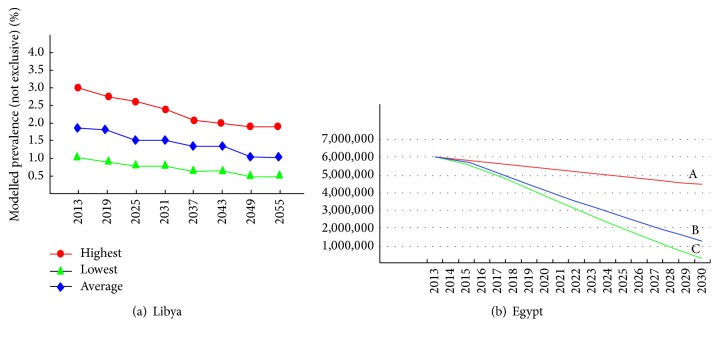
The impact of prevention and treatment on future prevalence rates of HCV. Modeled prevalence of hepatitis C virus infection Libya and Egypt. A: number of HCV-infected cases. B: number of cases after prevention. C: number of cases after prevention and treatment.

Though the rates of HCV infection are declining, particularly in Libya and Egypt, the decline has not yet met the expectations. Forecast studies are rarely done in North Africa. In Libya, Daw and his collaborators designed a mathematical model based on data collected from blood donors to predict the future status of HCV in the country [77]. The data show a stationary phase of HCV prevalence in the coming ten years. If preventive measures are applied, the prevalence will become very low within 50 years (Figure 3(a)). In Egypt, strategies have been built to increase diagnosis and treatment. Coupled with measures to prevent transmission, the result would be to control the disease and markedly reduce the prevalence and burden of HCV in the country by 2030 (Figure 3(b)) [78].

## 7. Conclusion

North African countries face a serious hidden crisis of HCV infections complicated by ignorance and inefficiency of public healthcare services and coupled with lack of research studies and programs for monitoring HCV infection. Countries such as Mauritania, Algeria, and Sudan should expand their efforts to address the emerging HCV epidemic among their populations. A multifaceted approach is needed. Regional and national guidelines for screening, treating, and preventing HCV infection should be endorsed and adopted by healthcare authorities and providers. Infected individuals and those who are at a higher risk should be provided with easy access to healthcare services. Implementation of advanced research and strengthening the practices of data collection and reporting of HCV infections should be given priority in the region of North Africa [79, 80].

## Figures and Tables

**Table 1:** Prevalence of HCV, development parameters, and genotype distribution in North African countries.

	Maghreb region					Nile region	
Countries	Libya	Tunis	Algeria	Morocco	Mauritania	Egypt	Sudan
Population (millions)	6.42	11	35	32	3.5	82.5	44.6
Population density (people/km^2^)	04	71	16	76	04	90	22
Literacy rate (M; F)	99.95% (99.97; 99.93)	98.06% (98.35; 97.76)	95.59% (95.65; 95.52)	83.19% (90.10; 75.87)	62.63% (70.04; 54.98)	91.12% (93.42; 88.73)	89.57% (91.29; 87.81)
GNI (PPP US$)	16020	3720	4420	2770	2400	6120	2120
HDI (2010–2014)	0.849	0.712	0.736	0.628	0.453	0.644	0.408
Country classification	HI	LMI	LMI	LMI	LI	LMI	LI

*Prevalence of HCV genotypes & subtypes*							
1	35%	67%	89%	68%	?	5%	5%
1a	1a	1a	1a	1a		—	—
1b	1b	1b	1b	1b		—	—
2	14.2	13%	9%	30%	?	2%	3%
2a	2a	2a	2a, 2b	2i, 2k		—	—
3	15%	3%	1%	—	?	—	—
4	29.2%	21%	1%	—	?	80%	90%
4a	4a	4a	4a	—		4a	4a
4k	4k	4k	4c, 4d	—	?	4k	—
							—
5	0.2%	—	—	—	?	—	—

GNI, gross national income. PPP, purchasing power parity. HDI, human development index. HI, high income. LMI, lower middle income. LI, low income.
